# Synergy of extreme drought and shrub invasion reduce ecosystem functioning and resilience in water-limited climates

**DOI:** 10.1038/srep15110

**Published:** 2015-10-13

**Authors:** Maria C. Caldeira, Xavier Lecomte, Teresa S. David, Joaquim G. Pinto, Miguel N. Bugalho, Christiane Werner

**Affiliations:** 1 Agroecosystem Research, BayCEER, University Bayreuth, Universitätsstr. 30, 95447 Bayreuth, Germany; 2 CEF, Instituto Superior de Agronomia, Universidade de Lisboa, Tapada da Ajuda, 1349-017 Lisbon, Portugal; 3 Instituto Nacional de Investigação Agrária e Veterinária I.P., Quinta do Marquês, Av, da República, 2780-159 Oeiras, Portugal; 4 Department of Meteorology, University of Reading, Earley Gate, PO Box 243, Reading RG6 6BB, United Kingdom; 5 Institute for Geophysics and Meteorology, University of Cologne, Pohlingstr. 3, 50969 Cologne, Germany; 6 CEABN-Inbio, Instituto Superior de Agronomia, Universidade de Lisboa, Tapada da Ajuda, 1349-017 Lisbon, Portugal; 7 Institute of Ecosystem Physiology, University Freiburg, Georges-Köhler-Allee 53/54, 79110 Freiburg, Germany

## Abstract

Extreme drought events and plant invasions are major drivers of global change that can critically affect ecosystem functioning and alter ecosystem-atmosphere exchange. Invaders are expanding worldwide and extreme drought events are projected to increase in frequency and intensity. However, very little is known on how these drivers may interact to affect the functioning and resilience of ecosystems to extreme events. Using a manipulative shrub removal experiment and the co-occurrence of an extreme drought event (2011/2012) in a Mediterranean woodland, we show that native shrub invasion and extreme drought synergistically reduced ecosystem transpiration and the resilience of key-stone oak tree species. Ecosystem transpiration was dominated by the water use of the invasive shrub *Cistus ladanifer*, which further increased after the extreme drought event. Meanwhile, the transpiration of key-stone tree species decreased, indicating a competitive advantage in favour of the invader. Our results suggest that in Mediterranean-type climates the invasion of water spending species and projected recurrent extreme drought events may synergistically cause critical drought tolerance thresholds of key-stone tree species to be surpassed, corroborating observed higher tree mortality in the invaded ecosystems. Ultimately, this may shift seasonally water limited ecosystems into less desirable alternative states dominated by water spending invasive shrubs.

Several regions of the world are experiencing recurrent extreme drought events which are projected to increase in intensity and frequency^1^,^2^. Extreme drought events alter ecosystem processes, including the hydrological cycle, plant productivity and delivery of ecosystem services^3^,^4^,^5^. Increased tree mortality, in particular, has been attributed to extreme and recurrent droughts especially in water-limited regions^6^, such as Mediterranean-type climate. Drought decreases soil water availability, induces stomatal closure in plants, reduces plant transpiration and carbon assimilation, ultimately leading to plant death^6^,^7^. However, plants diverge in their susceptibility to drought through species-specific water use strategies^7^,^8^, thus avoiding to surpass critical physiological thresholds for survival^9^. Simultaneously, plant invasion is increasing, affecting ecosystem functioning worldwide^10^,^11^,^12^,^13^. In particular, invasion either by native (shrub encroachment) or exotic woody plants in water-limited ecosystems can disturb the ecosystem water balance^11^,^14^,^15^ with potential implications for the hydrological cycle in these areas^16^. Shrub invasion and extreme drought events may interact, and further reduce soil water availability^12^ either directly through increased precipitation interception, or indirectly through enhanced water loss if invaders with water spending traits colonize the system^11^,^17^. This is particularly critical in water-limited ecosystems where evapotranspiration can equal or exceed annual precipitation input^3^,^18^, particularly under extreme drought events. In such cases, vegetation changes can rapidly induce a shift towards negative ecosystem water balance^3^. However, experimental evidence on the synergetic effects of shrub invasion and extreme events in ecosystems is lacking^12^. Understanding how these major drivers interact to affect the functioning and resilience^19^ of ecosystems is critical to predict responses to global change and implement effective ecosystem management and mitigation strategies^1^. We present the first study showing the synergistic effects of native shrub invasion and an extreme drought event. We used a Mediterranean cork-oak (*Quercus suber* L.) water-limited savannah-type ecosystem invaded by a native shrub (*Cistus ladanifer* L.) as a model system, and implemented an invader-removal experiment. These oak ecosystems, covering approximately 2.5 million ha in North Africa and Europe, have high conservation and economic value and are maintained by human use^20^. Cork-oaks are drought avoiding^8^,^21^ key-stone species in these open woodlands determining ecosystem functioning and ecosystem services delivery^20^. *C*. *ladanifer* encroachment in the absence of management is part of the ecological succession. However, in the last decades, most probably due to environmental and land use changes, it has rapidly expanded^22^,^23^,^24^ in the drier regions showing invasive patterns (“native invader”)^25^ forming dense mono-layered canopies and dominating the systems. This shrub is characterized by a water spending strategy and high growth rates^26^,^27^,^28^. Indeed, cork-oak mortality has been increasing and has also been associated with shrub encroachment by *C*. *ladanifer*^23^. Our objective was to investigate and quantify the interaction between shrub invasion and a co-occurring extreme drought event on tree and ecosystem transpiration and resilience. After a first year of baseline measurements, we implemented a shrub removal experiment and monitored soil moisture and plant and ecosystem water relations and fluxes during a three year period (see Methods) comprising an extreme drought event in 2011/2012 in Iberia (Fig. 1). We hypothesised that the water spending shrub invader should be more affected under extreme drought than the drought avoiding trees but that the synergistic effect of extreme drought and invasion would negatively impact ecosystem functioning.

Severe dry years in a Mediterranean environment are primarily characterised by a lack of winter precipitation, which is the rainiest season. The strong negative precipitation anomaly in 2011/2012 was observed all over Southwest Europe^29^, but affected primarily Iberia, where annual precipitation was regionally below 300 mm (Supplementary Fig. S1). The winter precipitation (December to February) was as low as 12.4% of long-term mean over the southwest Iberian Peninsula (Fig. 1a). Such extremely low winter precipitation is very uncommon in a Mediterranean-type climate, and was associated with exceptional large-scale atmospheric flow conditions over the North Atlantic and Europe^30^: the eddy-driven jet stream and rain-producing cyclones were shifted northward, associated with the recurrence of strong and persistent atmospheric ridges west of Iberia (see also Supplementary Discussion). The extreme dry winter was followed by a moderate and severe drought in spring and summer (about 71% and 7.5% of long-term mean precipitation respectively; Supplementary Fig. S1 and Supplementary Discussion). 2011/2012 was in fact the second driest year since 1950 (Fig. 1b), depicting precipitation deficits close to the driest year (2004/2005). Note that the extreme drought year of 2011/2012 was preceded and followed by years with slightly above average precipitation (Figs 1 and 2c).

The extreme drought strongly affected transpiration of both species and reduced total ecosystem transpiration by more than half (Fig. 2). Reduced winter and spring precipitation recharge resulted in very low soil water content (Fig. 3) and remarkably low stand transpiration during the main growing period in spring 2012 (Fig. 2b). Notably, invasive shrubs were the major contributors to total ecosystem transpiration in all three years (Fig. 2c), given their high population density (Supplementary Table S1) and high water use strategy^27^,^28^. In spite of being affected by extreme drought (52 ± 8% decline in transpiration), shrubs still reached higher maximum transpiration rates than trees in early spring (Fig. 2a). However, their high transpiration resulted in a rapid decline of leaf water potentials in summer (−3.92 ± 0.19 MPa, Supplementary Fig. S2). These semi-deciduous shrubs can effectively endure severe drought by shedding part of their leaves and rapidly resume growth when stress is released^27^,^28^. Furthermore, the high water consumption of the shrub invader exacerbated drought stress of trees. Tree transpiration in invaded stands declined much stronger (67 ± 13%) than that in stands cleared from invasive shrubs (31 ± 11%) relative to the pre-drought year (Fig. 2). Accordingly, trees in invaded stands had significantly (p < 0.05, n = 3) lower pre-dawn leaf water potential (−1.41 ± 0.08 MPa) than trees in uninvaded stands (−1.18 ± 0.05 MPa; for July, Supplementary Fig. S2).

There are large differences between species-specific critical thresholds beyond which extensive cavitation results in failure of the hydraulic transport system^9^. The invasive shrubs were able to endure much lower soil water potentials and drier soil conditions (Fig. 3, Supplementary Fig. S2) than trees as a result of lower critical cavitation thresholds^27^,^31^ (Supplementary Discussion). As oak trees have higher critical thresholds, they generally follow a drought avoiding, conservative water use strategy^21^, down-regulating stomatal transpiration (Fig. 2a) to avoid critical decline in leaf water potentials and detrimental runaway cavitation^9^,^31^. The effectiveness of this strategy is reflected in the rapid recovery of tree transpiration after the severe drought event in uninvaded stands (p < 0.05, n = 4; Fig. 4b). However, the significant reduction in water potential of trees in invaded stands diminished the tree hydraulic “safety margin”^9^ (Supplementary Fig. S2), which has recently been shown to be low for many tree species worldwide^9^. Consequently, trees in invaded stands were more vulnerable to hydraulic failure.

Overall soil moisture in the upper 100 cm diminished in the invaded stands which was particularly strong during recovery in spring 2013 (p < 0.01, n = 3; Fig. 3). Moreover, there was an interesting pattern in soil moisture distribution: the invaded stands showed a tendency of higher soil moisture in the upper 40 cm soil, where *Cistus* roots are most dominant^32^,^33^. This is in agreement with findings of a managed Portuguese cork-oak pasture, where the understory vegetation significantly increased rain infiltration in upper soil layers^34^. In contrast, shrub invaded stands showed a tendency of diminished soil moisture at deeper soil layers, which may be an indication of reduced percolation and lower deep soil water recharge (Fig. 3). Predawn water potentials, which reflect the soil water availability of the vegetation as the plants equilibrate with the soil overnight, further confirm the restricted water availability of the cork-oaks at invaded stands (Supplementary Fig. S2).

The impact of the synergistic effects of shrub invasion and extreme drought is most visible when comparing tree transpiration in invaded and uninvaded stands over the three years (Fig. 4a). The extreme drought event strongly reduced transpiration, which was more pronounced in trees in invaded stands. Resistance^35^, i.e. the capacity of trees to withstand severe drought, was not significantly different between stands (p > 0.05, n = 4; Fig. 4b), probably due to the overriding effect of the severe drought on trees. However, most remarkably, trees in invaded stands were not able to recover in the following wetter year maintaining lower transpirations rates, and showing lower resilience^35^ to the extreme drought (p < 0.05, n = 4; Fig. 4a), probably indicating severe damage of the hydraulic system^36^ and diminished access to deeper water pools^14^,^37^. The high density of shrubs in invaded stands with their dense shallow rooting system^32^,^33^ must have contributed to the drying out of the surface soil layers (Fig. 3) and could have resulted in a decreased deep soil moisture recharge, contributing to the lower recovery and resilience of the cork-oak trees in the invaded stands.

It has been suggested that drought-induced die-back of native plant species during extreme events may open temporary windows of opportunity for plant invaders^38^. Inversely, water-spending invaders have been expected to be more affected under extreme drought than drought-adapted natives^12^, which would lower their invasive potential. Our data do not support these hypotheses. The uninvaded system was well adapted to seasonal summer drought, and was able to withstand singular extreme events showing higher tree resilience and transpiration recovery than the invaded system (p < 0.05, n = 4; Fig. 4b). In contrast, tree transpiration in shrub invaded stands decreased from 23% to 16% (Fig. 2c), whereas invasive shrubs even increased their proportion of ecosystem transpiration from 77%, in the drought year to 84% in the post-drought year, indicating a further increase in competitive advantage of the invader. Further, the high water use by the invader diminished the difference between precipitation input and total transpiration losses in the extreme drought year, as compared to uninvaded ecosystem (66 mm and 248 mm, respectively in 2011/2012; Fig. 2c) which may drive these systems to the limit of sustainable water use. So far few studies have assessed the impact of invasion on water resources, but generally the effect on evapotranspiration was large when the invader exceeded the native vegetation in size and rooting depth (see recent review of Le Maitre *et al*.^16^). Our data further show that invasion and thickening of the shrub understory can also markedly alter water fluxes of the dominant tree species. Similar findings have been reported for the invasion of an exotic acacia invading the understorey of well-established Mediterranean forests thereby reducing pine tree transpiration by 25% albeit enhancing total ecosystem transpiration, pushing the margin of total water use close to annual precipitation input^11^.

Moreover, this is the first evidence quantifying the synergistic effects of an extreme event and plant invasion on the water balance of a natural open woody ecosystem, corroborating a significant lower resilience of the invaded ecosystem. We show that in agreement with the “fluctuating resource availability hypotheses”^39^, the shrub invader benefited from the decreased transpiration and resilience of the drought avoiding key-stone tree species due to the coupled negative effects of shrub invasion and extreme drought. We propose that ecosystem invasion by resource spending species may markedly increase variability and fluctuation of available resources in seasonally resource-limited ecosystem (Fig. 5). Given the occurrence of climatological extreme events, such variability may surpass critical cavitation thresholds of trees and impair their functioning (Fig. 5). The frequency of droughts like 2011/2012 affecting Southwestern Europe has increased in recent decades^1^,^29^. Furthermore, droughts are projected to further increase in frequency and intensity during the current century^1^,^2^. Therefore, the synergetic effects of drought and shrub invasion present an increasing threat for ecosystem functioning in the Mediterranean area and potentially also for other comparable regions of the globe.

Extreme events like the 2011/2012 drought have already caused large impacts on natural and agricultural ecosystems in many regions worldwide^4^,^6^. We suggest that, in water-limited ecosystems, the implications of our results can go beyond immediate effects on productivity and ecosystem functioning due to long-lasting effects through changes in species competitive balances in favour of water spending species. This may modify ecosystem-atmosphere exchange by altered carbon and water fluxes^4^,^11^,^18^, thus potentially changing regional climate feedbacks. Ultimately, this would increase tree mortality and shift savannah-type ecosystems into alternative states^19^,^40^ such as degraded shrublands^22^. Shrub invasions, combined with recurrent^41^ and projected increase of drought events, may decrease the resilience of water-limited ecosystems around the world and affect their functioning and delivery of ecosystem services.

## Methods

We established six 25 × 25 m randomly selected stands paired by 3 sites in a Mediterranean cork-oak (*Q. suber*) savannah-type ecosystem, invaded by the native shrub *C. ladanifer* (Supplementary Table S1). This shrub species forms dense and extended mono-specific stands. From February to November 2011 we conducted baseline measurements. In November 2011 all shrubs were cut in half of the paired stands, while the other half remained with intact shrub layer. All the aboveground shrub biomass was removed in the former. After shrub removal only a sparse annual herb layer remained, similar to the grassland understorey in naturally uninvaded areas, due to the extreme drought and grazing. All measurements continued until September 2013. Data presented in text are mean ± s.e.. We measured *in situ* meteorological data, monthly volumetric soil water content (4 access tubes per stand at 5 depths in 6 stands) and pre-dawn leaf water potential periodically (in 4 randomly selected trees and shrubs per stand in 6 stands). Sap flux density was measured continuously from 25^th^ of February 2011 to 30^th^ September 2013, in uninvaded and invaded stands, in 4 randomly selected trees and 4 shrubs per stand.

Sap flux density was measured in trees following the Granier constant heat method and the stem heat balance method in shrubs (see Supplementary Methods). Ecosystem transpiration (mm d^−1^) on a ground area and daily basis was determined by multiplying daily tree and shrub sap flux density by sapwood per ground area (m^2^ sapwood m^−2^ ground area) calculated for each species. Transpiration of the invaded ecosystem resulted from the sum of shrub and tree transpiration while transpiration of the uninvaded ecosystem resulted from tree transpiration. Transpiration of the herbaceous layer was not considered due to its low productivity. Sapwood per ground area was determined for *Q*. *suber* by measuring the DBH of all trees included in two circular plots with a radius of 14 m established randomly in each paired stands. For *C*. *ladanifer* shrubs we randomly established 12 plots of 4 m^2^ per site and measured the basal diameter of all shrubs. Allometric relationships between area of functional sapwood and DBH for *Q*. *suber* and basal diameter for *C*. *ladanifer* were used to determine total functional sapwood area (see Supplementary Methods, Table S1, S2 and S3 for further details). Volumetric soil water content was measured monthly with a vertical profiler PR1 at five depths (20, 30, 40, 60 and 100 cm) connected to the HH2 hand-held readout unit (Delta-T Devices, Cambridge, UK) in 4 access tubes randomly installed in each stand (4 tubes per stand, 2 stands per site, 3 sites).

We determined the resilience^19^,^35^ of tree transpiration to evaluate to which extent trees recovered acquiring pre-drought transpiration values. Resilience was calculated as the ratio of post-drought (2012/2013) to pre-drought^35^ (2010/2011) tree transpiration. Transpiration of trees in invaded stands during pre-drought year was used as baseline condition for calculating resilience. Resilience combines tree resistance to the extreme drought event and recovery after the extreme drought event^35^. Resistance was calculated as the difference between tree transpiration in the drought and the pre-drought year. Recovery was measured as the difference between tree transpiration in the post-drought and during drought year^35^.

Meteorological data were provided by the European Climate Assessment & Dataset project (ECA&D)^42^. Long term precipitation means (1950–2013) for the different seasons and full hydrological years (e.g., October 2011–September 2012) were computed. Precipitation anomalies are then built for each year relative to the long term mean. The daily evolution of cumulative precipitation deviations to the long term mean are analyzed for the area 10°W–5°W; 37°N–40°N (land areas only, mean 565 mm year^−1^). This comparison enables a visualization of the progression of the precipitation deficits (or exceedences) over the hydrological year on a daily basis.

### Data analyses

Univariate General Linear Models were used to test for differences in volumetric soil water content (SWC) among years (fixed factor) for each season (fixed), and site (fixed). Multivariate ANOVA (MANOVA) was used to test for effects of shrub removal on soil water content at different sampling dates as dependent variable (shrub removal, site and soil depth as fixed factors) and to test for differences in predawn leaf water potentials within sampling dates between shrubs and trees and between trees in invaded and uninvaded stands. Species (invasive shrub, uninvaded and invaded trees) as well as site were fixed factors. For multiple comparisons among treatments we used Bonferroni tests. Differences in resilience, resistance and recovery between trees in invaded and uninvaded stands were evaluated by a non-parametric Mann-Whitney Test. Requirements of normality and homogeneity of variance were tested when applicable. Statistical analyses were performed in SPSS (version 13, SPSS Inc., USA). Full Methods are available in the online version of the paper.

## Additional Information

**How to cite this article**: Caldeira, M. C. *et al*. Synergy of extreme drought and shrub invasion reduce ecosystem functioning and resilience in water-limited climates. *Sci. Rep*. **5**, 15110; doi: 10.1038/srep15110 (2015).

## Supplementary Material

Supplementary Information

## Figures and Tables

**Figure 1:**
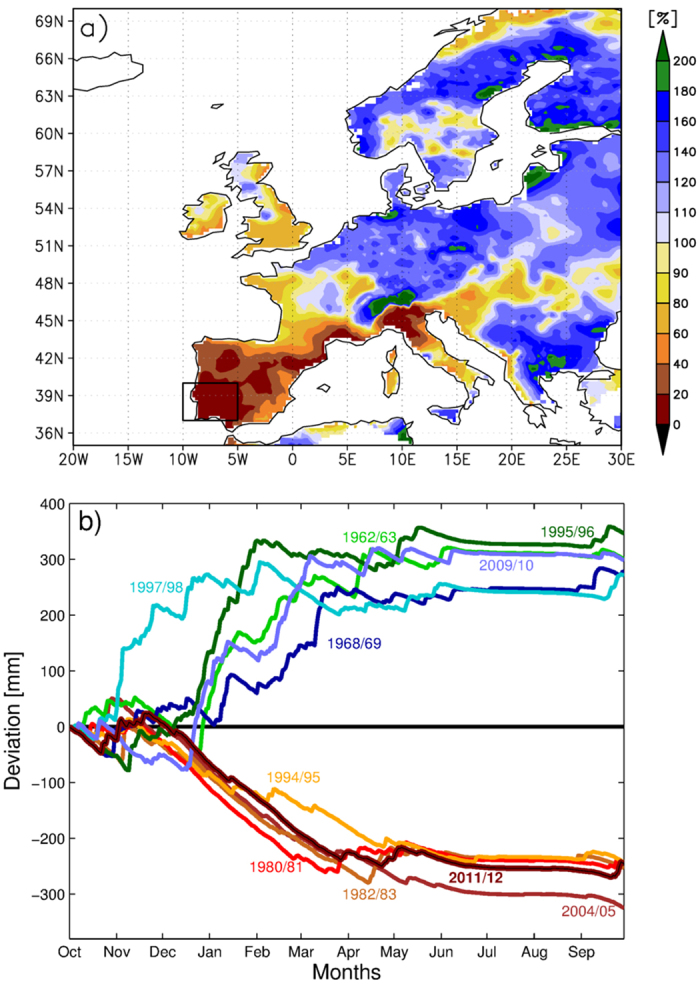
Precipitation anomalies during winter 2011/2012 over Europe, and cumulative precipitation anomalies for the five driest/wettest years for the southwest Iberian Peninsula since 1950. (**a**) Anomaly of winter (December 2011 to February 2012) relative to long-term mean for the period 1950-2013. 100% corresponds to long-term mean precipitation (565 mm); 20% corresponds to 20% of long-term mean precipitation during this period. (**b**) Accumulated precipitation deviations from climatology (1950–2013) during the hydrological year (1 October–30 September) for the area (10 °W–5 °W; 37 °N–40 °N) marked with a black box in (**a**). Only land grid points are considered.

**Figure 2:**
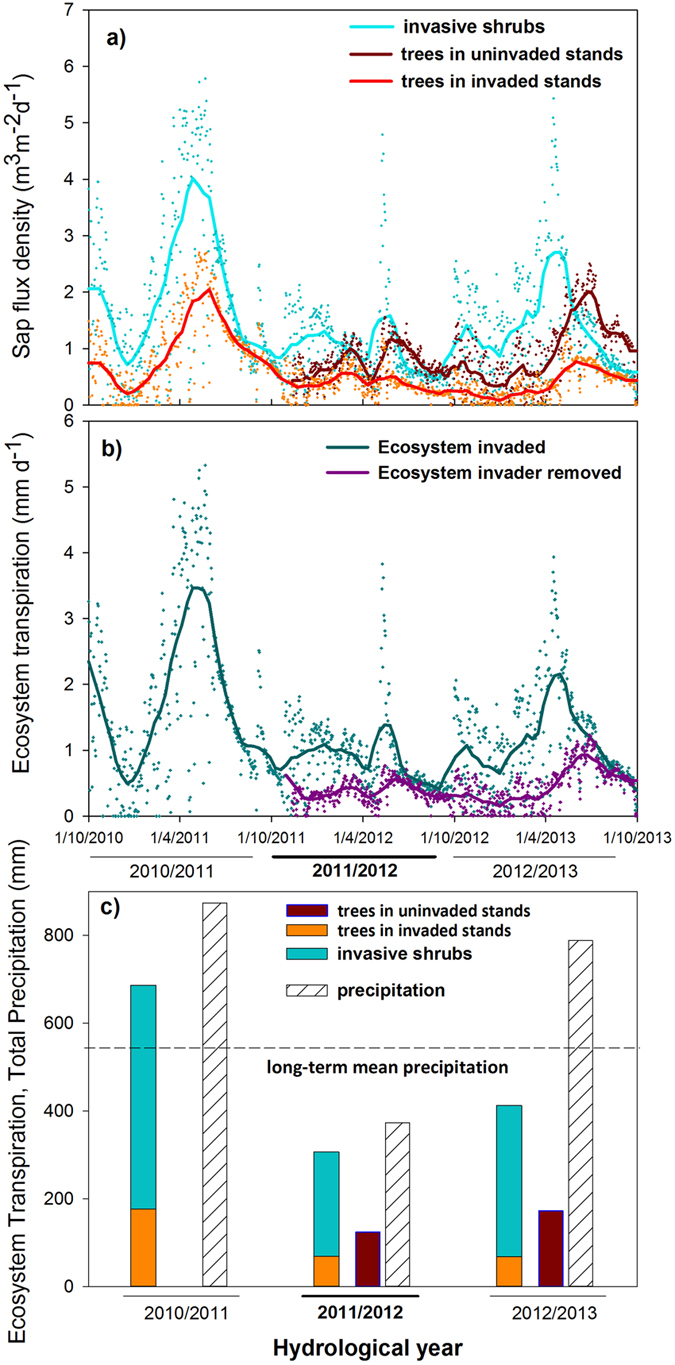
Effect of shrub invasion on ecosystem transpiration during the three hydrological years. (**a**) Daily sap flux density (i.e. water flux in the xylem) for the invasive *Cistus ladanifer* shrubs (cyan; n = 4); *Quercus suber* trees growing in shrub invaded stands (orange; n = 4) and trees after removal of the invader shrubs (uninvaded stands; n = 4) in Nov 2011 (dark red). (**b**) Ecosystem transpiration (mm d^−1^) for the invaded stands (green) and stands where the invader was removed (purple). Symbols mark daily sums, lines are best fit non-parametric smoothing kernel regression with a bandwidth of 0.25. (**c**) Precipitation and total annual ecosystem transpiration (mm y^−1^) for the invaded stands (stacked bars, equalling invasive shrub (cyan) and trees in invaded stands (orange) and for the uninvaded stands, where shrubs were removed in Nov 2011 (solely tree transpiration, dark red bars). Dash line indicates long-term mean precipitation.

**Figure 3:**
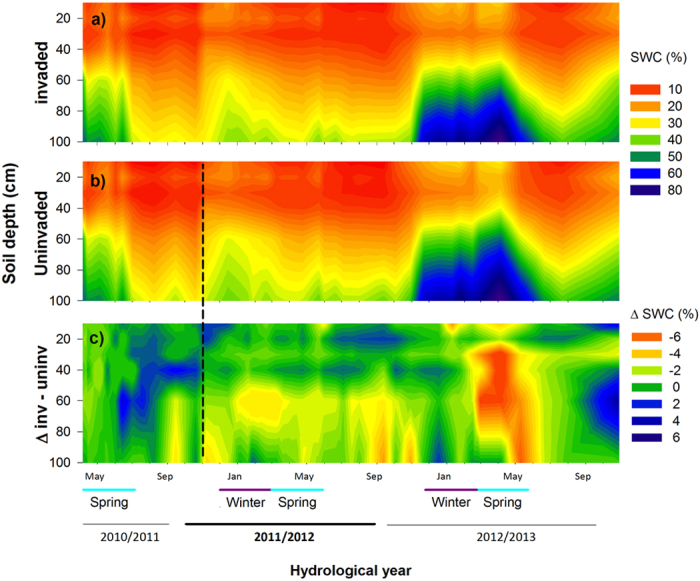
Changes in volumetric soil water content within the soil profile in invaded and uninvaded stands with time. Volumetric soil water content (SWC %; 4 tubes per stand, six stands; n = 3) at 5 depths (20, 40, 60, 80, 100 cm) from 2011–2013 at (**a**), *Quercus suber* stands invaded by shrubs and, (**b**) *Q. suber* stands where invading shrubs were removed in November 2011 (dashed line). (**c**) Difference (ΔSWC) in volumetric soil water content between invaded and uninvaded stands, with orange (negative differences) indicating drier and blue (positive differences) wetter soil at invaded stands. Multivariate analysis of variance (MANOVA) was used to test SWC differences along time after shrubs were cut, considering site and soil depth as fixed factors. Uninvaded stands had higher SWC than invaded stands (Wilks Lambda = 0.492, F_29,66_ = 2.352, p < 0.01; η^2^_p_ = 0.508). The interaction between treatment x time was significant (Wilks Lambda = 0.512, F_28,67_ = 2.284, p < 0.01; η^2^_p_ = 0.488). There was a significant interaction time x soil depth (Wilks Lambda = 0.001, F_140,335.83_ = 7.91, p < 0.001; η^2^_p_ = 0.763). The interaction time x treatments x soil depth was not significant (Wilks Lambda = 0.141, F_140,335.83_ = 1.170, p > 0.05; η^2^_p_ = 0.324).

**Figure 4:**
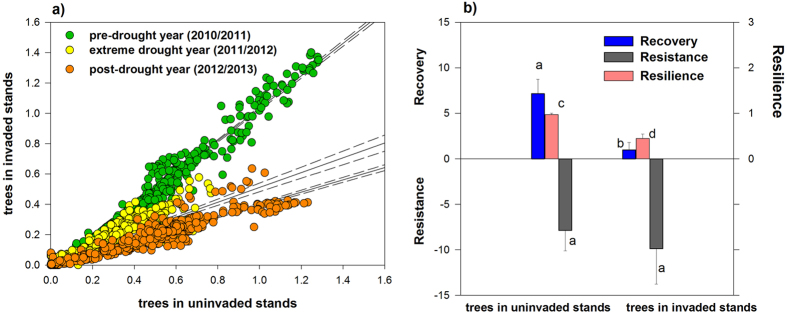
Relation between tree transpiration in invaded and uninvaded stands during the three hydrological years and resilience, resistance and recovery from extreme drought. (**a**) Green, yellow and orange symbols represent tree transpiration (mm day^−1^) in the pre-drought, extreme drought, and post-drought year. All regression lines were significant (p < 0.001 for all; r^2^_adj_ of 0.97, 0.59, 0.86, for pre-drought, drought and post-drought years, respectively), and 95% confidence intervals are given (dotted lines) (**b**), Tree resilience, resistance (m^3^) and recovery (m^3^) after an extreme drought in trees in uninvaded and invaded stands. Tree resistance of zero represents the maximum resistance of trees to the extreme event. Different letters denotes differences at p < 0.05 between invaded and uninvaded stands (non-parametric Mann-Whitney U Tests, n = 4).

**Figure 5:**
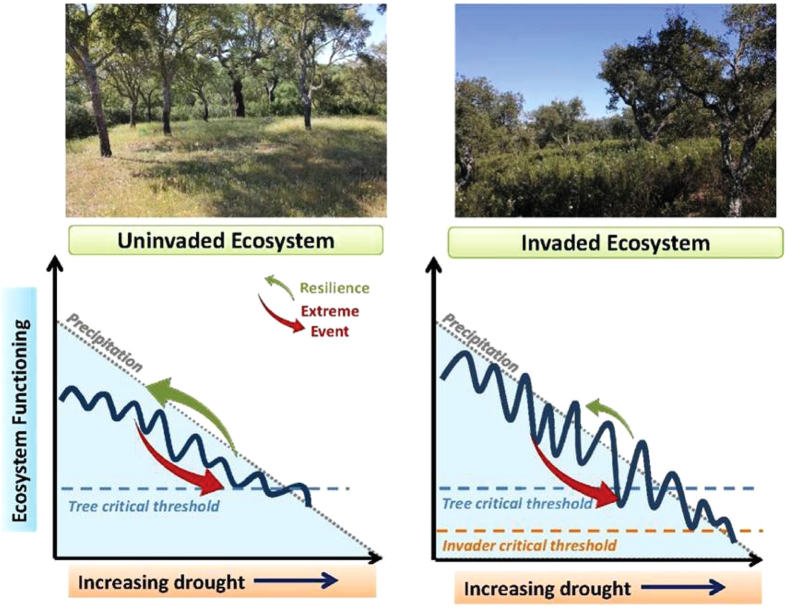
Conceptual figure of ecosystem response to synergistic effects of invasion and extreme drought coupled with the forecasted increasing dryness. Grey dash lines indicate forecasted decreasing trend in precipitation. Dark blue lines represent ecosystem transpiration, reflecting the variability of yearly water use. Lighter blue and orange dashed lines indicate the critical cavitation threshold for trees and shrubs, respectively. Red arrows represent an extreme drought event that can shift the system near critical limits of functioning. Resilience to extreme drought (green arrows) is higher in uninvaded systems and lower in systems invaded by water spending shrub species. Shrubs will enhance the amplitude and variability of ecosystem transpiration and gain competitive advantage over trees. If shrub invasion and extreme drought decrease water availability below the critical hydraulic thresholds for tree functioning, the resilience of the key-stone tree species will decrease and eventually lead to tree death. A critical transition point can turn open savannah-type of ecosystems into alternative states of degraded shrublands. Photographs by M C Caldeira.
