# Supplementary material for: Synergy of extreme drought and shrub invasion reduce ecosystem functioning and resilience in water-limited climates
Source: Sci Rep. 2015 Oct 13;5:15110. doi: 10.1038/srep15110 (PMC4602198; doi:10.1038/srep15110)
Supplement: Supplementary Information [file srep15110-s1.pdf]

Synergy of extreme drought and shrub invasion reduce ecosystem functioning and resilience in water-limited climates

Maria C. Caldeira, Xavier Lecomte, Teresa S. David, Joaquim G. Pinto, Miguel N. Bugalho, Christiane Werner

SUPPLEMENTARY METHODS

Study area. Our study was conducted in a cork-oak (*Quercus suber*) savannah-type ecosystem, located in a 900 ha estate in Portugal (38°47′N, 7°22′W). The site is invaded by a monospecific understorey of the native shrub *Cistus ladanifer* L.. *Q. suber* trees are approximately 50 years old while the monospecific *C. ladanifer* layer is approximately 10 years old. The density of trees is of 160 ± 18.62 trees per ha and of invader shrubs 21667 ± 2602 shrubs per ha (Table S1). Shrubs were cut in November 2011 in half of the stands. Because *C. ladanifer* is an obligate seeder and does not resprout from roots, shrub removal was effectively implemented through a single cutting. Climate is Mediterranean-type, with temperate winters and precipitation falling mainly in the winter half-year, and with hot and dry summers. Soils are shallow with a schist bedrock bellow.

Table S1| *Q. suber* savannah-type ecosystem characteristics. Diameter is at breast height (DBH) for trees and at ground level (basal) for *C. ladanifer* shrubs. Data are means ± s.e.

	Quercus suber	*Cistus ladanifer*
Density (number individuals ha^{-1})	160 ± 18.62	21667 ± 2602
Diameter (cm)	46.71 ± 5.51	15.94 ± 0.42
Sapwood/ground area (m^2 m^{-2})	0.00049 ± 7.28E^{-5}	0.00068 ± 3.67E^{-5}

The ecosystem. Cork-oak ecosystems have a relatively low tree density of approximately 60 to 180 trees per ha, forming a structurally savannah-type habitat composed by an evergreen oak canopy cover and an understory of a variety of shrub species intermixed with diverse annual C3 grasslands. These ecosystems have high conservation and economic value and are maintained by human use[20]. Cork-oak trees are mainly managed and exploited for their bark - cork - a non-wood forest product, which is harvested each 9 to 12 years without killing the tree. Cattle grazing can also occur and, more seldom, agricultural crops. Beyond provisioning ecosystem services such as cork and livestock, they also generate regulating services such as long-term carbon storage and fire prevention services. Human use maintains a mosaic of land-uses and habitat types with high conservation value[20]. Lack of human use, drought and wildfires are increasingly turning these ecosystems into shrublands, dominated by shrubs such as *Cistus* spp[22,23]. Simultaneously, tree mortality has also been increasing[23].

Field measurements. A weather station was located in an open area adjacent to the experimental site. Data was measured continuously and half-hourly data stored in a data logger (DL2e, Delta-T Devices Ltd, Cambridge, UK). Vapour pressure deficit (D, kPa) was computed from 30 min averaged relative humidity and air temperature. Volumetric soil water content was measured monthly with a vertical profiler PR1 at five depths (20, 30, 40, 60 and 100 cm) connected to the HH2 hand-held readout unit (Delta-T Devices, Cambridge, UK) in 4 access tubes randomly installed in each stand (4 tubes per stand, 2 stands per site, 3 sites). Tree water status was estimated by measuring predawn leaf water potentials using a Scholander-type pressure chamber (PMS 1000, PMS Instruments, Corvalis, Oregon) in fully expanded *Q. suber* leaves (n=3-4, per tree) (4 trees per stand) and twigs (n=2-4) of *C. ladanifer* shrubs (4 shrubs per

stand). *Q. suber* leaves were taken at the same height in the canopy. Measurements were performed on 17 dates along the 3 years of the experiment.

Sap flow of 4 trees in invaded stands, 4 trees in shrub released (designated as "uninvaded") stands and 4 shrubs (invaded stands) were measured from the 25th of February 2011 to the 30th September 2013 in 2 randomly chosen stands (invaded and uninvaded). The Granier constant heat method[43-45] was used to estimate sap flux density of trees. Stem heat balance method[46-49] was used in shrubs as their small stem diameters prevented the use of needle probes. A summary of both methods is presented here but theoretical basis and full methodology is given in the latter references. All sensors were protected from rain and heat with plastic bubble wrap and covered with aluminium reflectors. Missing daily fluxes values due to episodic probe failure were gap filled using the best fit linear regressions (based on adjusted r^2 and residuals distribution) against functioning sensors. From October 2010 to February 2011 daily sap flux density of trees and shrubs was derived from stepwise multiple regressions on daily sap flux density and PAR and D during non-limiting soil moisture conditions (adjusted $r^2 > 0.90$, $p < 0.001$ for all regressions; Table S2). Sap flow sensors were replaced whenever malfunctioning was detected.

Table S2| Regression coefficients for prediction of daily sap flux density for *Q. suber* and *C. ladanifer* from October 2010 to February 2011 based on daily accumulated photosynthetic active radiation (PAR) and daily average vapour pressure deficit (D), during non-limiting soil moisture conditions. Regressions were highly significant ($p < 0.0001$).

Species	constant	PAR	D	$r^2_{adjusted}$
Quercus suber	- 0.325	0.000071	0.400	0.91
Cistus ladanifer	- 0.486	0.000163	1.083	0.90

One sensor, with 30 mm long probes (TDP30 sensors, Dynamax, Texas, USA), was installed radially and at breast height in the northwest side of the trunk of each tree, to avoid direct sun exposure and heating. Sampled trees had a diameter at breast height (DBH) of 45.86 cm ± 4.9 (mean ± s.e.), covering the range of tree DBH distribution in the stand (Table S1). Sap flow sensors measured the temperature differential between a paired heated and unheated thermocouples every minute and were recorded as means of 30 min in a data logger (CR1000 and AM16/32 multiplexer, Campbell Scientific, Logan, UT, USA). Sap flux density was estimated using the empirical relationship determined by Granier[43-45], considering 10-day moving average of the maximum temperature differential at which sap flow is zero.

Daily sap flux density was computed to avoid stem capacitance effects[50]. Total sap flow of each tree was determined by scaling the sap flux density by the whole cross-sectional area of the functional sapwood. The depth of functional sapwood was determined by wood coring 10 trees at breast height and measuring sapwood depth based on immediate visual assessment of coloration of sapwood. An allometric relationship between functional sapwood area and DBH (cm) was determined (Table S3). The functional sapwood depth of all the measured trees was greater than 30 mm and was assumed as uniform over the entire depth. This approach has given reliable estimates of sap flux data for *Q. suber* trees in previous experiments[21].

Table S3| Allometric equations between sapwood area (A_s) and diameter at breast height (DAP) for *Q. suber* trees and basal diameter (d) for *C. ladanifer* shrubs

Species	Allometric equation	$r^2_{adjusted}$
Quercus suber	$A_s = 0.1624 \times DAP^{1.1822}$	0.92
Cistus ladanifer	$A_s = 0.3739 \times d^{2.1173}$	0.71

Sap flow gauges (SGA13, Dynamax, Texas, USA) were fitted to shrubs with average diameter of 1.59 ± 0.054 (mean ± s.e.). The sap flow gauges consist on a heater tape mounted on a flexible cork sheet that is wrapped around the stem of the shrub and is covered with insulation material. A constant heating power was supplied to the heater. Pairs of thermocouples measure the temperature gradients associated with conductive heat losses up and down the stem, and radially through the cork sheet. The heat balance equation to compute the sap flux rate is derived from the energy input from the heater and the temperature gradients. The gauge outputs were measured every minute and averaged every 30 minutes in a datalogger (CR1000 and AM16/32 multiplexer, Campbell Scientific, Logan, UT, USA). To minimize the influence of environmental thermal gradients and rain, gauges were fixed at least at 40 cm above the soil surface and were wrapped in several layers of aluminum foil. Positions of sap flow gauges were changed during spring and summer to prevent stem damage due to heating. Functional sapwood area was determined by cutting 20 stems of shrubs that were immersed in water with a red food dye (Vahiné, France) and put under the sun to transpire. After 12 hours the dyed sapwood area was measured. Based on this data, an allometric regression between basal diameter and functional sapwood area was constructed for *C. ladanifer* shrubs (Table S3).

Meteorological data. Meteorological data were provided by the European Climate Assessment & Dataset project (ECA&D)[42]. The E-OBS dataset was produced by the EU-FP6 ENSEMBLES project, is regularly updated and incorporates information from available precipitation datasets into gridded daily precipitation fields for Europe on a grid of 0.25° latitude × 0.25° longitude. The period used in this study is 1950 - 2013. The daily gridded precipitation data was used to estimate long term annual and seasonal mean precipitation for 1950-2013, from which anomalies for 2011/2012 and other years were derived. Hydrological years (e.g., starting for example in 1. October 2011 and ending in 30. September 2012) are considered. Additionally, the evolution of cumulative precipitation deviations along the hydrological year are compared to the long term mean within the area 10°W – 5°W; 37°N -40°N on a daily basis (land areas only). The long-term mean annual precipitation in this area is 565 mm. This comparison enables a visualization of the progression of the precipitation deficits (or exceedences) over time and thus for individual seasons / months.

Discussion on meteorological conditions during 2011/2012. Mediterranean type climates are characterized by a large seasonality of precipitation, which occurs primarily in winter, while in summers hardly any precipitation falls. As most precipitation occurs in winter, dry years are primarily characterized by a lack of winter precipitation[51,52]. The total annual precipitation in the hydrological year of 2011/2012 was below 600 mm over most of Iberia, in some areas below 300 mm (Fig. S1a). Over Southwestern Iberia, annual precipitation was roughly half of long-term average for 1950 - 2013 (56.6 %, 320 mm from 565mm, Fig. S1b). For the winter (December - February), precipitation anomaly was 12.4 % (28.8 mm, long-term average 232.3 mm) for Southwestern Iberia (Fig. 1a). The precipitation anomaly for spring (March – May) over Iberia was about 71 % (moderate drought, 98.1 mm from 138.2 mm, Fig.

S1c), followed by a severe drought in summer (June – August) with hardly any precipitation (1.7 mm from 22.7 mm, Fig. S1d). The daily evolution of precipitation deficits for Southwest Iberia is shown in Figure 1b. After near average precipitation during October and November, the precipitation deficits become evident from early December, maintaining a strong deviation from climatology for the rest of the hydrological year. Severe precipitation deficits in summer affected whole Southern Europe, particularly the Mediterranean area roughly up to 45°N (fig. S1d). The hydrological year of 2011/2012 was the second driest year recorded since 1950 (Fig. 1B), after the driest record in 2004/2005[51,53]. The strong precipitation anomalies in the winter 2011/2012 over Southwestern Europe were associated with very anomalous large-scale atmospheric flow conditions over the North Atlantic and Europe[30]. These include a northward shifted upper-level polar jet stream and cyclone tracks over the North Atlantic, associated with a very positive phase of the North Atlantic Oscillation[54], and the recurrent occurrence of strong and persistent upper-level atmospheric ridges west of Iberia[55], which effectively induced the very low precipitation over Southwestern Europe. There is evidence that the return period of droughts like 2011/2012 has shortened in recent decades[29].

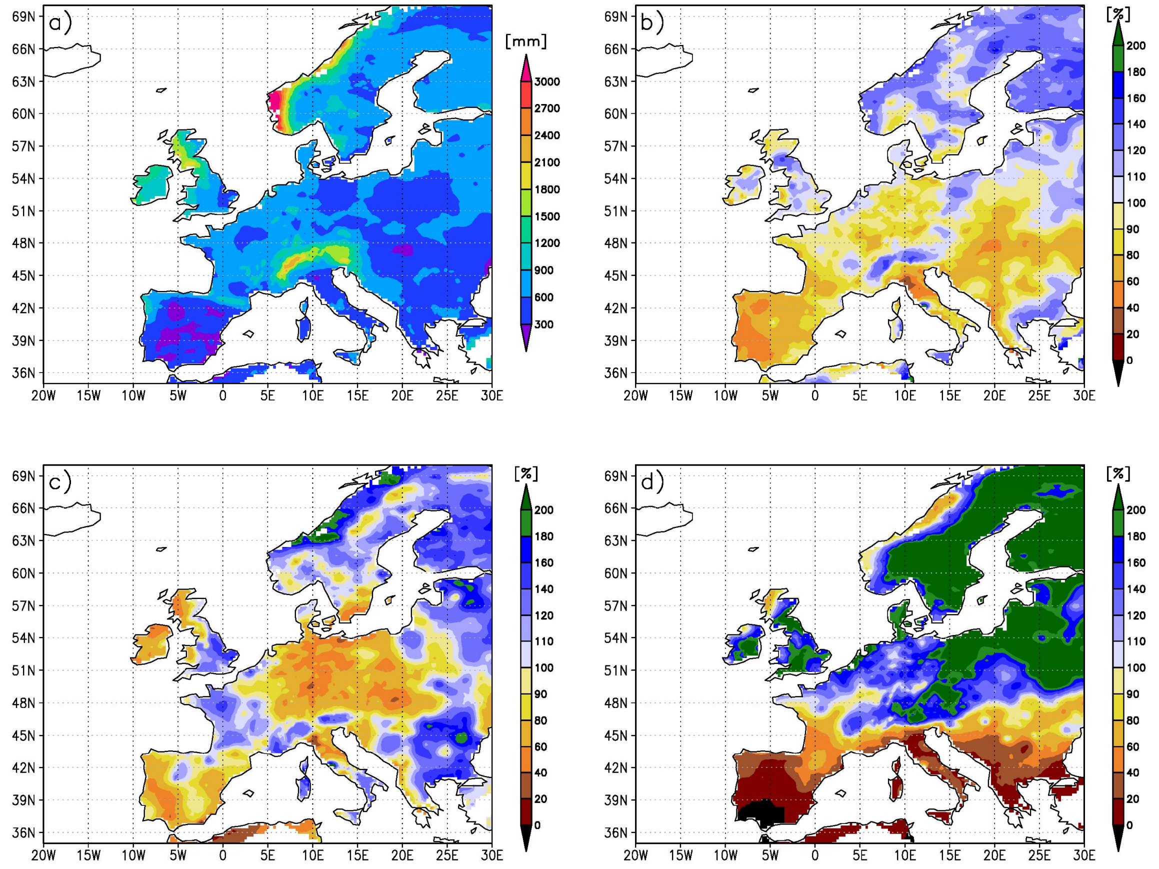

Figure S1| Annual precipitation in hydrological year 2011/2012 over Europe and precipitation anomalies for different periods during 2011/2012. a), Total annual precipitation (mm) for the hydrological year (October 2011 – September 2012). **b),** anomaly for annual precipitation compared to climatology 1950-2013. **c),** as **b)** but for spring 2012 (March – May). **d),** as **b)** but for summer 2012 (June – August). 100% corresponds to average precipitation; 20% corresponds to 20 % of long-term mean precipitation during this period. For more details please see Supplementary Discussion.

Discussion on effect of drought and invasion on water relations. Tree and shrubs species have different water use strategy and drought tolerance traits. Evergreen Mediterranean oaks have a

conservative water use strategy[21,26], although their deep root system can enable access to deeper ground water reserves[56] they down-regulate stomatal conductance and up-regulate photoprotective mechanisms upon drought to protect their long-lived leaves[57,58]. They operate at lower transpiration rates but throughout a longer period of the season compared to shrubs. However, this strategy can be counter effective if trees are growing in competition with water spending shrubs[26], which exhibited higher transpirations rates, which occurred earlier in spring in all years (Fig. 2). Due to their water-spending strategy leaf water potentials of shrubs were always lower than that of trees ($p < 0.001$) and declined rapidly during the extreme drought year (Fig. S2).

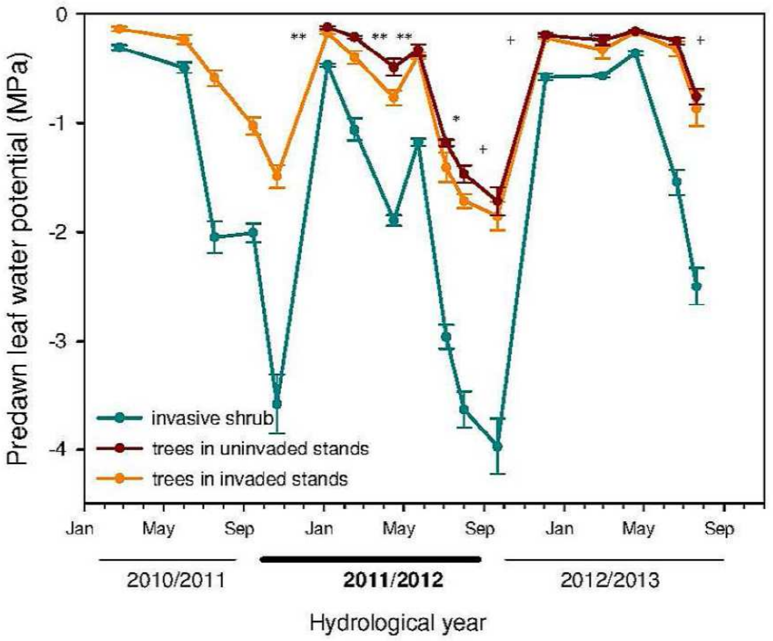

Figure S2 | Tree and shrub predawn leaf water potentials (Ψ_{pd}). Data are means ± s.e. (n = 3 stands; 4 individuals per species per stand) for invader *C. ladanifer* (dark cyan symbols), *Q. suber* trees in invaded stands (orange symbols) and *Q. suber* trees after removal of the invader

shrub in Nov 2011 (trees in uninvaded stands, dark red symbols). Ψ_{pd} were significantly different between invader shrubs and trees overall dates (MANOVA, Wilks Lambda = 0.004, $F_{16,11}$ = 163.59, p < 0.001; η^2_p = 0.99). Average Ψ_{pd} of shrub invader (-1.80 ± 0.04) was significantly lower for all sampling dates (Bonferroni comparisons p <0.001 for all sampling dates) than that of trees (-0.69 ± 0.02). The interaction time x species was significant (Wilks Lambda = 0.010, $F_{15,12}$ = 78.92, p < 0.001; η^2_p=0.99) suggesting a faster decrease of Ψ_{pd} of shrub invader as compared to trees. There were no significant interactions between time and treatment (Wilks Lambda = 0.28, $F_{12,6}$ = 1.27, p = 0.40; η^2_p=0.72). Predawn water potential of trees in invaded plots was significantly lower than that of trees in uninvaded plots ($F_{1, 17}$ = 12.33, p = 0.003). Bonferroni comparisons were performed to assess differences between dates (+ denotes p < 0.7, * denotes p < 0.05 and ** denotes p < 0.01).

The faster decrease of Ψ_{pd} of the shrubs as compared to trees was also reflected in a significant time x species interaction (p < 0.001; Fig. S2). Soil water content in winter and spring of the extreme drought year was significantly lower than during the preceding and following wetter year (p < 0.001; Fig. 3). The high water consumption by the shrub invader together with the extreme drought further reduced soil water availability (Fig. 3) and trees were unable to maintain their water status (Fig S2). In general, when soil water availability decreases below critical levels, plants regulate water loss to avoid damaging runaway cavitation. However, there are large differences between species-specific thresholds when extensive cavitation results into failure of the hydraulic transport system[9]. Such threshold, defined as water potentials at 50% loss of xylem conductivity (Ψ_{50}), is twice as low for *C. ladanifer* (-6.2 MPa)[27] compared to the cork-

oak trees (-2.9 MPa)[31]. Trees in invaded stands had significantly lower leaf water potential than trees without shrub competition (Fig. S2). This diminishes the hydraulic safety margin (i.e. $\Psi_{minimum}$ - Ψ_{50})[9], i.e. the difference between the minimum leaf water potentials and Ψ_{50}, within which cork-oak trees functions. Moreover, cavitation thresholds can be different for different organs in the plants. Leaves or fine roots can have lower cavitation thresholds originating leaf drop or hydraulic isolation of fine roots[36] promoting tree survival in the short-term but impairing tree recover when soil water becomes available again. The lower water potentials of trees at the invaded sites most probably induced severe cavitation damage, as the trees in invaded stands were not able to recover during the wet year to pre-drought transpiration levels in contrast with trees in uninvaded stands (Fig. 4). Slopes of the linear regressions between tree transpiration in the invaded and uninvaded stands (Fig. 4a) were different among the three years (95 % confidence interval does not include 0) and decreased from 1.07 in the pre-drought year to 0.41 in the post-drought year showing higher tree transpiration in the uninvaded stands in the post-drought year. Thus, while trees in uninvaded stands were able to recover from severe drought, the synergistic effect of invasion and drought did shift the cork-oaks to the critical thresholds resulting in strongly reduced resilience of this key-stone trees species (see conceptual Fig. 5).

Additional references:

43. Granier, A. Une nouvelle méthode pour la mesure du flux de sève brute dans le tronc des arbres. *Ann. Sci. For.* **42**, 193-200 (1985).

44. Granier, A. Evaluation of transpiration in a Douglas-fir stand by means of sap flow measurements. *Tree Physiol.* **3**, 309-320 (1987).

45. Lu, P., Urban, L., Zhao, P. Granier´s thermal dissipation probe (TDP) method for measuring sap flow in trees: theory and practice. *Acta Botanica Sinica* **46**, 631-646 (2004).

46. Sakuratani, T. A heat balance method for measuring water flux in the stem of intact plants. *J. Agr. Meteorol.* **37**, 9-17 (1981).

47. Sakuratani, T. Improvement of the probe for measuring water flow rate in intact plants with the stem heat balance method. *J. Agr. Meteorol.* **40**, 273-277 (1984).

48. Baker, J. M., van Bavel, C.H.M. Measurement of mass flow of water in the stems of herbaceous plants. *Plant, Cell Environ.* **10**, 777-782 (1987).

49. Steinberg, S. L., van Bavel, C.H.M., McFarland, M.J. A gauge to measure mass flow rate of sap in stems and trunks of woody plants. *J. Am. Soc. Hortic. Sci.* **114**, 466-472 (1989).

50. Oren, R. and Pataki, D.E. Transpiration in response to variation in microclimate and soil moisture in southeastern deciduous forests. *Oecologia* **127**, 549-559 (2001).

51. García-Herrera, R. D. *et al*. The outstanding 2004-2005 drought in the Iberian Peninsula: The associated atmospheric circulation. *J. Hydrometeor*. **8**, 483–498 (2007).

52. Santos J. A., *et al*. The role of large-scale eddies in the occurrence of precipitation deficits in Portugal. *Int. J. Climatol.* **29**, 1493-1507 (2009).

53. Santos, J. *et al*. Atmospheric large-scale dynamics during the 2004/2005 winter drought in Portugal. *Int. J. Climatol.* **27**, 571–586 (2007).

54. Wanner, H. *et al*. North Atlantic oscillation. Concepts and studies. *Surv. Geophys.* **22**, 321–382 (2001).

55. Santos, J. A. *et al*. On the development of strong ridge episodes over the eastern North Atlantic. *Geophys. Res. Lett.* **36**, L17804 (2009).

56. Kurz-Besson, C. *et al*. Hydraulic lift in cork oak trees in a savannah-type Mediterranean ecosystem and its contribution to the local water balance. *Plant and Soil* **282**, 361–378 (2006).

57. Werner, C. & Correia, O. Photoinhibition in cork-oak leaves under stress: influence of the bark-stripping on the chlorophyll fluorescence emission in *Quercus suber* L. *Trees* **10**, 288-292 (1996).

58. Werner, C., Correia, O. & Beyschlag, W. Characteristic patterns of chronic and dynamic photoinhibition of different functional groups in a Mediterranean ecosystem. *Funct. Plant Biol.* **29**, 999-1011 (2002).
